# Altered Expression of Antimicrobial Peptides in the Upper Gastrointestinal Tract of Patients with Diabetes Mellitus

**DOI:** 10.3390/nu15030754

**Published:** 2023-02-02

**Authors:** Oliver Linn, Bernhard Menges, Frank Lammert, Susanne N. Weber, Marcin Krawczyk

**Affiliations:** 1 Department of Medicine II, Saarland University Medical Center, 66421 Homburg, Germany; 2 Department of Medicine I, Marienhausklinikum St. Elisabeth, 66740 Saarlouis, Germany; 3 Hannover Medical School (MHH), 30625 Hannover, Germany; 4 Laboratory of Metabolic Liver Diseases, Department of General, Transplant and Liver Surgery, Medical University of Warsaw, 02-091 Warsaw, Poland

**Keywords:** AMP, cathelicidin, defensin, insulin resistance, LL-37

## Abstract

Antimicrobial peptides (AMP) are essential components of innate immunity with a broad range of antimicrobial activities against bacteria, viruses, and fungi. The aim of this study was to investigate AMP expression in the upper gastrointestinal tract in normal and pathological metabolic states in humans. Furthermore, we examined the correlation between vitamin D levels and AMP expression in the same cohort. Serum concentrations of 25-hydroxyvitamin D3 were measured, and mRNA expression of β-defensins HBD-1, -2, -3, -4, α-defensins HD-5 and -6 and cathelicidin in the upper gastrointestinal tract epithelia were determined by quantitative RT-PCR in 31 individuals (10 with type 2 diabetes, 10 with insulin resistance, and 11 healthy controls). The majority of the cohort showed low vitamin D concentrations, which were negatively correlated with mRNA expression levels of HBD-3 in corpus mucosa. *HBD-1* and *HBD-3* mRNA were expressed in corpus mucosa, with the former significantly decreased in patients with diabetes. Hence, we conclude that type 2 diabetes is associated with reduced AMP expression in the upper gastrointestinal tract, which might contribute towards epithelial barrier dysfunction and increased bacterial translocation in these patients.

## 1. Introduction

Human AMPs can be divided into three main groups: α-defensins, β-defensins, and cathelicidin. These are cationic, amphipatic peptides, 29–47 amino acids in length and 3–5 kDa in weight with a β-sheet structure. α- and β-defensins are defined by the location of specific disulfide bonds. The α-defensins Human Defensin 5 (HD-5) and -6 (HD-6) are expressed in the Paneth cells of the upper gastrointestinal tract. These cells are exocrine serous glandular cells at the base of small intestinal crypts in the duodenum, jejunum, and the terminal ileum. Human β-defensins 1 to -4 (HBD-1, -2, -3, -4) are expressed in various epithelial tissues. The third main AMP found in humans is the cationic antimicrobial peptide 18 (hCAP18), the only human cathelicidin. Discovered in 1995, it is also referred to as LL-37. HCAP18/LL-37 is expressed in the epithelium of the esophagus, stomach, small intestine, and colon [1,2].

AMPs exert broad antimicrobial activity, achieved by disruption of cell membranes, protein biosynthesis and the transcription and translation machinery of pathogenic microorganisms, and by activating other sections of the immune system. The main mechanism of AMP function is the formation of voltage-dependent, ion-permeable channels, and thereby the permeabilization of cell membranes of microorganisms. Subsequently, DNA-, RNA- and protein-synthesis of the targeted microorganism are inhibited by disturbance of the transmembrane transport processes, resulting in the elimination of osmotic and voltage gradients and a loss of metabolic substrates. To mediate these effects, AMPs require a gradient between positively charged AMPs and the negatively charged outer cell membrane of the microorganism in order to destroy it. This explains the lack of activity of AMPs against unenveloped viruses and against human cells, which have a neutral total charge.

AMPs act chemotactically to attract T-helper cells and dendritic cells as well as to activate the complement system. They show a broad spectrum of activity against Gram-negative and Gram-positive bacteria, fungi, viruses, and protozoa [3,4]. There is also recent evidence for the activity of AMPs against the SARS-CoV-2 virus [5]. Recent studies explore the modulation of wound healing, angiogenesis and carcinogenesis by defensins, as well as the use of defensins as biomarkers and therapeutic agents to improve the integrity of the mucosal barrier [6,7].

In healthy individuals, AMP expression is influenced by acute or chronic infections and pathogen contacts. Several studies have outlined the details of AMP gene expression in the upper gastrointestinal tract [8,9,10,11]. There is consensus that the β-defensin HBD-1 is expressed constitutively throughout the upper gastrointestinal tract independently of pathogen exposure. All other β-defensins (HBD-2, -3 and -4) show only moderate or no expression [2,12]. 

Of note, specific receptor and signal cascades regulating AMP expression were identified in *Drosophila melanogaster* [13,14,15,16]. Here, the metabolic state influences AMP expression. In human cell lines, AMP expression was induced by starvation in vitro [17,18]. The effect of a diabetic metabolic state on AMP expression was first investigated in a rodent model. Type 2 diabetes was associated with reduced expression of β-defensin in the kidney, and insulin administration normalized its expression [19]. AMP expression is known to be affected by vitamin D: The human vitamin D receptor VDR is a transcription factor involved in the regulation of the cathelicidin/LL-37-encoding gene *CAMP*, as well as *DEFB4,* the gene encoding HBD-2 [20,21].

Our hypothesis is that a hyperglycemic metabolic setting of diabetic patients, or even the prodromal state of insulin resistance, leads to suppression of AMP expression. Therefore, the specific aim of this study was to determine the relative mRNA expression of *HBD-1, HBD-2, HBD-3, HBD-4, HD-5, HD-6* and *cathelicidin/LL-37* in biopsies of the upper gastrointestinal tract from patients with type 2 diabetes or insulin resistance. Furthermore, a possible correlation between vitamin D status and AMP expression was investigated. 

## 2. Patients and Methods

Patients were recruited during elective, outpatient or inpatient esophagogastroduodenoscopies (EGD) at the Caritas-Klinik in Dillingen, Germany, and the Department of Medicine II, Saarland University Medical Center, Homburg, Germany. Informed consent was obtained from all subjects involved in the study. Ethical approval was granted through the ethics committee of the Ärztekammer des Saarlandes (protocol code 108/11) on 13 July 2011.

The study subjects presented with multiple pre-existing gastrointestinal symptoms or pre-existing diabetes mellitus type 2 (T2DM), which was either untreated or treated with oral antidiabetic agents, insulin, or both. Both symptomatic (diabetic side effects or long-term organ dysfunction) and asymptomatic diabetic patients were included. 

Anthropometric data, pre-existing disorders, especially T2DM, pre-existing treatment with antidiabetic drugs, proton pump inhibitors, or non-steroidal anti-inflammatory drugs, and pre-existing vitamin D supplementation were documented. Blood samples were obtained prior to EGD and analyzed for fasting serum glucose and insulin concentrations, HbA1c and serum 25-hydroxyvitamin D3 levels. Insulin resistance was calculated using the HOMA-IR-model (Homeostasis Model Assessment-Insulin Resistance) [22,23]. If necessary, an oral glucose tolerance test (2 h OGTT) was performed in individuals presenting with capillary fasting glucose levels between 100 mg% and 126 mg%. All patients were fasted for at least 12 h at the time of examination. 

Laboratory results were used to stratify the patients into three groups:

- Group 1: Patients with inadequately controlled manifest diabetes mellitus (pathological fasting blood glucose levels > 126 mg% and HbA1c > 7.5%) or pathological OGTT (whole blood glucose capillary ≥ 200 mg%).
- Group 2: Patients with insulin resistance (HOMA index > 2.5 and euglycaemic metabolic state, HbA1c value < 6.5%).
- Group 3: Metabolic healthy subjects as controls (fasting blood glucose levels < 100 mg%, HbA1c < 5.8%, HOMA index ≤ 1).

During EGD, biopsies in the upper gastrointestinal tract were obtained in the duodenal bulb, gastric antrum, gastric corpus and middle esophagus for mRNA isolation. Biopsies were immediately deep frozen in liquid nitrogen and stored at −70 °C. Two further tissue samples were taken in the stomach antrum and corpus to detect a possible colonization with *Helicobacter pylori* by commercial rapid urease tests.

RNA was isolated from the obtained biopsies (Qiagen RNeasy^®^ mini kit, Qiagen, Hilden, Germany). Using spectrophotometry, the concentration and purity of the isolated RNA was determined. A reverse transcription PCR (RT-PCR) was performed, and cDNA complementary to the isolated mRNA was synthesized. Then, the synthesized cDNA was amplified by PCR. Quantitative real-time PCR to determine the relative amount of mRNA, corresponding the expression of the target gene, was performed on the TaqMan^®^ PCR system 7500 Fast (Applied Biosystems^®^ Life Technologies, Darmstadt, Germany). Relative expression of target mRNAs was calculated by the delta-delta-Ct-method [24] using GAPDH for normalization. The mRNA extraction and expression measurements could be performed in all 31 patients. An overview of the examined target genes and their gene expression assays is shown in Table 1. Please refer to Supplementary Materials (Tables S1–S7) for further information on all performed PCR procedures. 

To compare the demographic data and baseline characteristics of included patients, as well as the relative AMP gene expression between groups, we performed Fischer’s exact test and non-parametric single-factor analysis of variance (ANOVA) according to Kruskal–Wallis, as appropriate. The relative gene expression at the distinct sites (esophagus, gastric corpus, gastric antrum, duodenum) was compared between the control group, the insulin resistance group, and the diabetes group. The significance level was set at *p* < 0.05. Mann–Whitney U-tests were performed as post hoc tests, and the significance level threshold was corrected for multiple testing according to Bonferroni and set to *p* < 0.017. Vitamin D serum concentration and relative AMP expression were compared using the Spearman rank correlation coefficient. The extent of the correlation was investigated exploratively for all AMPs in esophagus, gastric corpus, gastric antrum, and duodenum.

## 3. Results

### 3.1. Patient Cohort

The study cohort (n = 31, 21 female) was divided into three groups: a control group (n = 11), a group with insulin resistance (n = 10), and a group with manifest diabetes mellitus (n = 10). 

There were no significant differences between groups in age, gender distribution and height (Table 2). There were also no differences in the number of patients with pre-existing vitamin D supplementation (one patient), basal vitamin D levels, or the frequency of pre-existing treatment with proton pump inhibitors (16 patients) or non-steroidal anti-inflammatory drugs (8 patients). Only two patients in the control group presented with esophagitis, less than in the insulin resistance group (7 patients) or the diabetes group (7 patients). There were no significant differences in the number of patients suffering from gastritis or duodenitis with respect to the number of *Helicobacter pylori*-positive patients. 

The body weight of the patients with insulin resistance (95.90 ± 15.77 kg; *p* = 0.002) or diabetes (85.90 ± 24.18 kg; *p* = 0.039) was significantly higher than in the control group (65.64 ± 8.66 kg). The body mass indices of the insulin resistance group (34.92 ± 5.03 kg/m^2^; *p* < 0.001) and the diabetic group (31.69 ± 5.69 kg/m^2^; *p* < 0.001) were significantly higher in comparison to controls (23.03 ± 2.96 kg/m^2^). Fasting glucose concentrations (126.40 ± 10.43 mg%), capillary blood glucose (169.11 ± 81.66 mg%) and HbA1c levels (8.82 ± 2.53%) in the group of patients with diabetes were significantly higher than those in the control group (glucose 91.73 ± 11.33 mg%; and 98.82 ± 7.82 mg%, respectively; *p* < 0.01; HbA1c 5.60 ± 0.29%; *p* < 0.001).

The insulin resistance group and the control group differed significantly (*p* = 0.002) with respect to fasting insulin levels: in the insulin resistance and the control group, the corresponding values were 20.51 ± 14.33 ng/L and 3.13 ± 1.53 ng/L, respectively. With regard to the HOMA index, there was also a significant (*p* = 0.012) difference between the insulin resistance group (5.88 ± 4.93) and the control group (1.05 ± 0.75). Two patients showed capillary glucose levels between 100 mg% and 126 mg% in the initial blood glucose measurement and underwent an oral glucose tolerance testing, which came up as normal. The patients were stratified to the insulin resistance group due to their pathological HOMA indices and a history of diabetes mellitus. 

Table 2 provides an overview of all patient characteristics and summarizes the group differences. 

### 3.2. RNA Isolation and Gel Electrophoresis

The amount of RNA per sample was determined by spectrophotometry. Only samples that reached an RNA concentration of >200 ng/µL with a high RNA purity (OD 260/280 ≥ 2.0) were included. 

### 3.3. AMP Expression in the Upper Gastrointestinal Tract

**Figure 1:**
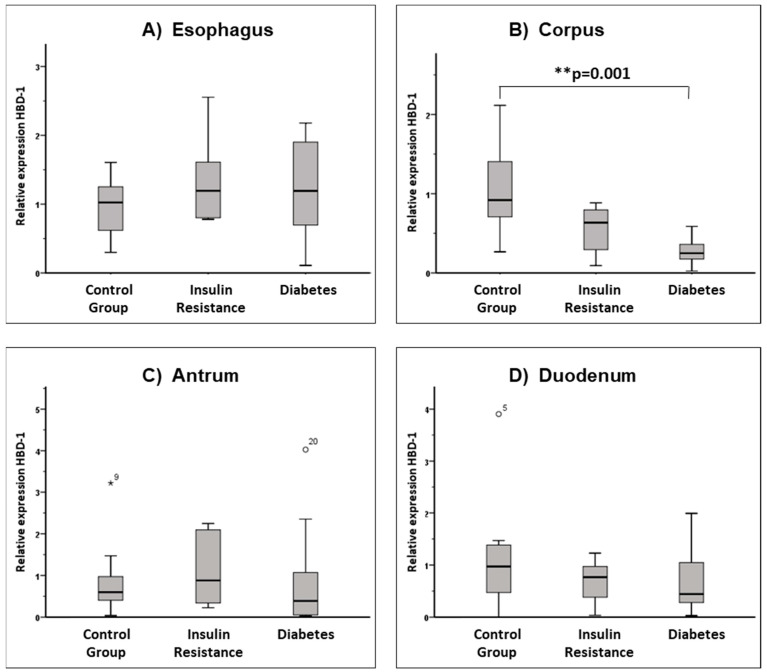
Relative expression of *HBD1* in the upper gastrointestinal tract. Box whisker plots presenting relative mRNA expression of *HBD1* in the mucosal biopsies of the three groups studied: control group (n = 11), insulin resistance (n = 10) and diabetes group (n = 10) in the locations (**A**) esophagus, (**B**) corpus, (**C**) antrum, and (**D**) duodenum. In the control group, *HBD1* mRNA could be detected in the esophagus in 10 patients, and in the gastral corpus, antrum and duodenum in 11 patients. In the insulin resistance group and the diabetes group, *HBD1* mRNA was detected in all patients in all samples studied. There is a significant (*p* = 0.001) difference in expression in the corpus between the controls and patients with diabetes. Far outliers have been marked with a star ⋆^9^, outliers with a circle: ○^20^, ○^5^. The superscripted number indicates which observation in the data set is the outlier.

A total of 124 biopsies were obtained. *HBD1* mRNA was detected in 123 (>99%) of biopsies. Significant expression differences existed for HBD-1 in the gastric corpus. Both global expression differences between the groups (*p* = 0.002) and a significant reduction in *HBD1* expression in the gastric corpus in the diabetes group as compared to the control group (*p* = 0.001) could be detected (Figure 1A–D).

### 3.4. Correlation of Vitamin D Levels with AMP Expression

The mean 25-hydroxyvitamin D3 concentration in the serum samples of 31 patients was 16.7 ± 9.5 ng/mL. The control group (n = 11) presented a mean level of 16.9 ± 8.3 ng/mL, and the corresponding levels in the insulin resistance (n = 10) and T2DM groups were 16.0 ± 7.8 ng/mL and 17.2 ± 12.8 ng/mL, respectively. There were no significant differences in vitamin D levels between the three groups. In total, thirteen patients (42%) showed vitamin D levels <20 ng/mL, eight patients (26%) had levels <10 ng/mL, and one patient had a vitamin D level below the detection limit (<5 ng/mL). Only one patient received vitamin D supplementation (vitamin D level 26.6 ng/mL). 

**Figure 2:**
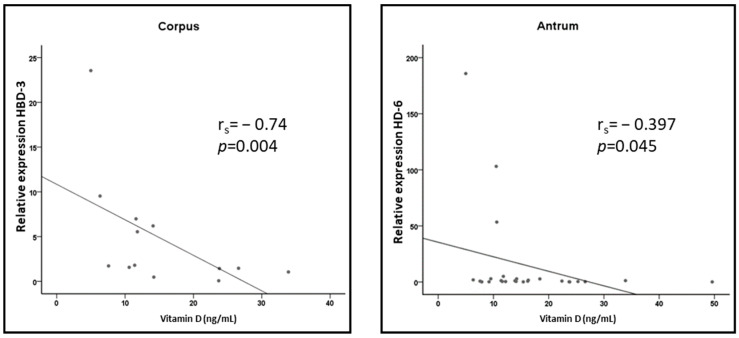
Scatter plot illustrating the correlations of vitamin D levels with the expression of *HBD3* in the gastric corpus and *HD6* expression in the gastric antrum.

In the gastric corpus, we detected a significant (*p* = 0.004) negative correlation of vitamin D levels with the expression of *HBD3* (r_S_ = −0.74) (Figure 2). In the gastric antrum, a non-significant (*p* = 0.045) negative correlation of vitamin D levels with the expression of *HD6* could be observed (r_S_ = −0.397). There was no correlation in any other location, and none of the other AMPs showed a relevant negative or positive correlation between their expression levels and the vitamin D levels in the upper gastrointestinal tract (all *p* > 0.05).

There was a significant negative correlation between vitamin D levels and the relative mRNA expression of *HBD3* in gastric corpus (r_s_ = −0.74; *p* = 0.004) and a trend for *HD6* in gastric antrum (r_s_ = −0.397; *p* = 0.045).

## 4. Discussion

In this study, we quantified the relative mRNA expression of antimicrobial peptides HBD-1, HBD-2, HBD-3, HBD-4, HD-5, HD-6 and cathelicidin/LL-37 in the upper gastrointestinal tract of patients with type 2 diabetes mellitus. The aim of the study was to investigate a possible link between AMP expression and glucose homeostasis in humans. In addition, a possible correlation of AMP expression levels with vitamin D serum concentrations was scrutinized.

The study cohort of patients with available clinical data and AMP gene expression levels in the upper gastrointestinal tract comprised 31 patients, who were divided into three groups. Esophagitis was more common in the insulin resistance and diabetes groups than in controls, in agreement with previous studies reporting that high fasting glucose levels and high HOMA indices are associated with an increased prevalence of erosive esophagitis and gastroesophageal reflux disease (GERD) [25]. Diabetes mellitus type 2 and high HbA1c have been associated with an increase in pathological endoscopy findings in the esophagus [26]. One explanation for this observation is that diabetic gastroparesis delays the stomach emptying and, thus, may promote the development of GERD. Diabetic angiopathy might also reduce the blood flow through the mucosa, resulting in impaired epithelial resistance to pathogens and the development of ulcers.

*Helicobacter pylori* was diagnosed in four patients of the diabetes group, and the relative expression of *HBD-2* was increased in these patients in the gastric corpus and antrum. This elevation was not significantly different from *HBD-2* mRNA expression in patients with negative *Helicobacter pylori* status. In other studies, *Helicobacter pylori*-positive gastritis resulted in significantly increased gastric expression of HBD-2, HBD-3, HBD-4, and LL-37. PPI-therapy was reported to have no influence on the expression of HBD-2, HBD-4, or LL-37 [27,28].

Of all AMPs examined, significant expression differences between the groups were detected for HBD-1 only. In the gastric corpus, HBD-1 expression was significantly lower in the T2DM group than in the control group. Previous studies have shown HBD-1 expression in vivo only in patients with *Helicobacter pylori*-positive or -negative gastritis or in healthy individuals, but not in patients with diabetes [27,29]. These studies were able to demonstrate constitutive HBD-1 expression in healthy subjects throughout the upper gastrointestinal tract. Hence, it was assumed that HBD-1, unlike other AMPs, is not subject to significant expression variation in healthy subjects [10,11,14,27,29,30]. In patients with *Helicobacter pylori*-positive gastritis, the studies reported contradictory results with respect to HBD-1 expression. In *Helicobacter pylori*-positive gastritis, the induction of HBD-1 expression was detected [29]. Other studies revealed either no relevant overexpression [27] or reduced expression of HBD-1 [31]. In this study, four out of ten patients with type 2 diabetes, but no patient in the control group, were positive for *Helicobacter pylori*. HBD-1-expression was previously examined in patients with diabetes mellitus type 1 and 2. When investigating polymorphisms of the *DEFB1* gene (Human β-defensin 1 is encoded by the *DEFB1* gene, in this study also referred to as *HBD1)*, the genotype [GG] of SNP c.44C>G was less frequently detected in patients with diabetes and might be associated with reduced *HBD-1* expression in peripheral blood [32].

Other AMPs measured in this study showed no significant differences in expression between groups along the entire upper gastrointestinal tract. *HBD-2* mRNA was detected in the entire upper gastrointestinal tract in all groups with the lowest absolute detection signals in duodenum. Except for one study that did not find relevant gastric or duodenal *HBD-2* expression, *HBD-2* has been detected previously in the entire upper gastrointestinal tract. *HBD-3* expression has been described in the esophagus, gastric fundus, gastric antrum and duodenum, but expression in the gastric corpus has to date not been reported in healthy subjects [2,12,27,28]. In the current study, *HBD-3* expression was found throughout the entire gastrointestinal tract and in the gastric corpus. There were no significant differences in expression between the groups, but a trend towards lower expression in the T2DM group was observed.

*HBD-4* mRNA was found in all groups throughout the entire gastrointestinal tract. At the same time, relative expression levels of *HBD-4* were the lowest of all AMPs examined in this study. In other studies, *HBD-4* mRNA was found inconsistently in the upper gastrointestinal tract, which may be due to low expression levels in healthy subjects and increased expression in gastritis. Previously, *HBD-4* expression has been described in the gastric corpus and antrum [28]. Only one study described robust expression levels of *HBD-4* in the esophagus and duodenum [2]. In the present study, *HBD-4* mRNA could be detected in 52% of all biopsies, most frequently in the duodenum.

The highest expression levels of *HD-5* and *HD-6* were observed in the duodenum and terminal ileum, where the density of the Paneth cells, which synthesize and store α-defensins, is highest [1,2]. In this study, *HD-5* and *HD-6* expressions were detected in the entire upper gastrointestinal tract in all groups. Our analysis showed the lowest expression in the gastric corpus, and the highest expression in the duodenum, which is in line with previous reports [1,2].

Cathelicidin/LL-37 has been shown to be inconsistently expressed in the upper gastrointestinal tract, with only low level expression in healthy subjects [11] and no detection in esophageal and corpus biopsies [2]. In the present study, *LL-37* expression could only be detected in 55% of the biopsies investigated. The lowest absolute detection rates were observed in gastric biopsies.

Our study outlines for the first time an effect of the diabetic metabolic state on AMP expression in the upper gastrointestinal tract in vivo in humans. Previous AMP expression studies examined metabolically healthy subjects with inflammatory diseases of the upper gastrointestinal tract. The relationship between AMP expression and diabetic metabolic state was previously reported in diabetic rats. Studies have shown reduced mRNA of rat *β-defensin-1* in the kidney tissue of overweight rats with type 2 diabetes, which was related to an increased susceptibility to infection. Stress- or corticoid-induced suppression of *β-defensin-3* in the esophagus was detected in a diabetic mouse model [19,33]. Stress increases the level of endogenous corticoids, which causes the same AMP-suppressing effect as external corticoid administration [34]. However, increased expression of *β-defensin-1* in the kidney tissue of diabetic rats has also been noted, and the contradicting results were explained by different stages of diabetic nephropathy [35]. At the onset of nephropathy, defensin expression is increased, possibly in response to or as protection against disease progression. HBD-1 shows the highest expression levels in the urogenital tract in humans, especially in kidney tissue, where it appears to be subject to marked expression fluctuations. Both reduced [36] and increased expression of *HBD-1*-mRNA were detected in human kidney cell lines (HEK) grown in increasing glucose concentrations [37,38]. Our findings may be supported by recent publications that showed decreased HBD-1, HBD-2 and HBD-3 levels in the saliva of patients with T2DM and decreased HBD-3 levels in saliva of patients with type 1 diabetes [39,40]. Mohanty et al. [41] detected decreased levels of the antimicrobial protein psoriasin in the plasma and urine exfoliated cells of patients with high blood glucose levels. Psoriasin was also decreased in human urothelial cell lines cultured under high glucose conditions and in the urinary bladder of type 2 diabetic mice, backing the hypothesis that a hyperglycemic metabolic state may compromise innate immunity. Besides decreased psoriasin levels, high glucose levels promote downregulation of IL-1β, IL-6 and occludin, as well as changes in the cytoskeleton that facilitate bacterial invasion. Interestingly, the same study showed no alteration of *HBD-1* and *HBD-3* expression levels in the urothelial cell lines under high glucose conditions. Conflicting reports on differential AMP expression are common, showing that the underlying mechanisms of AMP expression are not yet fully understood.

The mechanisms of humoral immune defense have been investigated in detail in the fruit fly *Drosophila*, which shows parallels to the innate immune defense in humans. Two AMP signaling pathways were identified, the Toll-like receptor (TLR) and the immune deficiency signal pathway (IMD) [13]. These signaling pathways are comparable to the Toll-like and tumor-necrosis factor-α receptor signaling pathways in humans, respectively, and both are triggered by pathogen contacts. Gene expression in response to inflammatory stimuli is coordinated by, among others, transcription factors of the NF-kΒ family [14,42]. Furthermore, it was shown in *Drosophila* that the insulin-like signaling pathway regulates not only growth and lifespan but also energy homeostasis by adapting the metabolism to food supply. The forkhead-box O-family (FOXO) of transcription factors regulates genes that directly affect energy metabolism [43,44,45]. FOXO has been shown to increase AMP expression independently of infection in response to stress and energy deficiency [17,18]. Insulin signaling, however, reduces the activity of FOXO. Thus, an excess of circulating glucose leads to suppression of FOXO-dependent AMP expression. We investigated and confirmed this hypothesis in the present study, at least for HBD-1: The previously postulated constitutive expression of *HBD-1* in the gastrointestinal tract appears to be modulated by the metabolic state, and reduced expression might contribute towards impaired mucosal barrier function in diabetes. In fact, increased fasting serum glucose levels due to insulin resistance might result in AMP repression prior to manifestation of T2DM. In bronchial cell culture lines, a high insulin concentration was able to inhibit the FOXO-mediated expression of HBD-2 [46].

In recent years, the relevance of 1,25-dihydroxyvitamin D3 as a regulatory factor of the immune system has been recognized [20,21]. Numerous studies have shown a connection between vitamin D and the expression of LL-37 and HBD-2 in vitro and in vivo. Vitamin D substitution is associated with higher AMP expression [47]. Vitamin D activates the vitamin D receptor (VDR), which binds to vitamin D response elements (VDRE) in the promoters of *HBD-2*, *LL-37* and numerous anti-inflammatory genes [20]. In this study, serum vitamin D concentrations were negatively correlated with *HBD-3* expression in the corpus and *HD-6* expression in the antrum, but the absolute *HBD-3* and *HD-6* expression levels were both very low. The other AMPs studied showed no correlation with serum vitamin D concentrations; in particular, a positive correlation between vitamin D and the expression of *HBD-2* and *LL-37* reported in previous studies could not be confirmed in our cohort. These results might be due to the low serum vitamin D levels in our cohort and the generally low AMP expression rates in the upper gastrointestinal tract. The reference and target range of serum vitamin D concentrations and the recommended daily intake doses are discussed controversially. However, serum vitamin D concentrations below 20 ng/mL define vitamin D deficiency in several current guidelines and recommendations [47,48].

There are several limitations to this study: Overall, the number of patients is low, with a mere 31 individuals from a cohort of 89 initially recruited participants. During the recruitment period, only patients with an indication to perform an EGD were included. Insufficiently treated diabetic patients with an HbA1c value > 7.5% are rarely diagnosed in everyday clinical practice and frequently present with comorbidities or were anticoagulated, which in our study setting ruled out a biopsy during elective EGD. 

After evaluating different housekeeping genes in preliminary experiments and experiencing relevant CT value variation, we selected *GAPDH* as a robust housekeeping gene in our study, as in previous studies on AMP expression in the upper gastrointestinal tract [9,27]. 

Upper gastrointestinal tract AMP expression has previously been investigated in the context of infections or in healthy individuals, but not in patients with T2DM. In this study, *HBD-1* expression in gastric corpus was reduced in the diabetic metabolic state. 

## 5. Conclusions

Considering an increased susceptibility of patients with diabetes to infections, our observation of reduced epithelial AMP expression might provide a mechanistic explanation for the impairment of the epithelial barrier defense, enabling bacterial overgrowth and translocation. The restoration of epithelial integrity through normalization or even induction of AMP expression may represent a future therapeutic target in metabolically induced intestinal barrier deficiency. Unfortunately, the chemical nature of AMPs is prohibitive to large scale production and widespread commercial use. Consisting of peptides, they are prone to proteolytic degradation, and production costs are high [5,49]. Given the rise of new infectious diseases such as COVID-19 and an increasing number of multi-resistant pathogens, there is an urgent need to translate the promising in vivo capabilities of AMPs into clinical applications. 

## Figures and Tables

**Table 1:** Gene expression assays.

Target Gene	Synonym	Assay Identification Number
*GAPDH*	*GAPDH*	Hs 02758991_g1
*HBD1*	*DEFB 1*	Hs 00608345_m1
*HBD2*	*DEFB 4*	Hs 00175474_m1
*HBD3*	*DEFB 103*	Hs 00218678_m1
*HBD4*	*DEFB 104*	Hs 00414476_m1
*HD5*	*DEFA 5*	Hs 00360716_m1
*HD6*	*DEFA 6*	Hs 00427001_m1
*LL-37*	*CAMP*	Hs 00189038_m1

Overview of the investigated target genes, their synonyms and their assay identification numbers.

**Table 2:** Baseline characteristics of the study cohorts.

	Control Group	Patients with Insulin Resistance	Patients with Diabetes	Total
Number	11	10	10	31
Age (years)	58 ± 16	68 ± 10	62 ± 14	63 ± 14
Gender (male/female)	3/8	3/7	4/6	10/21
Weight (kg)	65.64 ± 8.66	95.90 ± 15.77 **	85.90 ± 24.18 *	81.94 ± 21.04
Height (m)	1.69 ± 0.09	1.65 ± 0.05	1.64 ± 0.14	1.66 ± 0.1
BMI (kg/m^2^)	23.03 ± 2.96	34.92 ± 5.03 **	31.69 ± 5.69 **	29.66 ± 6.85
Pre-existing diabetes	0	1	8	9
Antidiabetic treatment (OAD/insulin/both)	0/0/0	0/0/0	6/2/10	6/2/10
Vitamin D supplementation	0	1	0	1
PPI therapy	4	7	5	16
NSAID therapy	2	4	2	8
Fasting glucose (mg%)	91.73 ± 11.33	111.50 ± 13.88	126.40 ± 10.43 **	106.00 ± 17.98
Capillary glucose (mg%)	98.82 ± 7.82	118.67 ± 15.22	169.11 ± 81.66 **	126.79 ± 53.82
HbA1c (%)	5.60 ± 0.29	5.97 ± 0.44	8.82 ± 2.53 **	6.76 ± 2.03
Insulin (ng/L)	3.13 ± 1.53	20.51 ± 14.33 **	12.42 ± 9.62	11.56 ± 12.48
HOMA index	1.05 ± 0.75	5.88 ± 4.93 *	3.77 ± 2.82	3.42 ± 3.93
Vitamin D (ng/mL)	16.9 ± 8.3	16.0 ± 7.8	17.2 ± 12.8	16.7 ± 9.5
Normal Mucosa (EGD)	6	2	1	9
Esophagitis	2	7 *	7 *	16
Corpus gastritis	1	1	4	6
Antrum gastritis	0	1	2	3
Duodenitis	2	0	0	2
*Helicobacter pylori* positive	0	0	4	4

Overview of absolute frequencies and mean values (± standard deviation). Abbreviations: HbA1c = glycated hemoglobin; HOMA = homeostatic model assessment; NSAIDs = non-steroidal anti-inflammatory drug; OAD = oral antidiabetic; PPI = proton pump inhibitor. Significant differences in comparison with controls are marked * (*p* < 0.05) or ** (*p* < 0.01).

## Data Availability

Patient data are part of the relevant minimal data set. The data of all patients who consented to share the data with other researchers are available upon request from the corresponding author, since the ethical committee demanded individual consent of patients whether or not patient data may be forwarded. Readers may contact the corresponding author to request the data.

## Supplementary Materials

The following supporting information can be downloaded at: https://www.mdpi.com/article/10.3390/nu15030754/s1, Tables S1–S7: Additional information on PCR-procedures.
