# Supplementary material for: Altered Expression of Antimicrobial Peptides in the Upper Gastrointestinal Tract of Patients with Diabetes Mellitus
Source: Nutrients. 2023 Feb 2;15(3):754. doi: 10.3390/nu15030754 (PMC9919831; doi:10.3390/nu15030754)
Supplement: Supplementary file 1 [file nutrients-15-00754-s001.zip › nutrients-2116384-SI.pdf]

Supplementary Tables

Table S1

Kits/Reagents and Disposables	Source
High capacity cDNA Reverse Transcription Kit	Applied Biosystems® Life Technologies, Darmstadt, Germany
DEPC-H_2O	Merck, Darmstadt, Germany
Ribonuclease-Inhibitor (RNase Out ™)	Invitrogen™ Life Technologies, Carlsbad, CA, USA
Reverse Transcriptase (MultiScribe™)	Applied Biosystems® Life Technologies, Darmstadt, Germany
Eppendorf tubes, pipettes, pipette tips	Eppendorf AG, Hamburg, Germany
Centrifuge (5415C, 5424R)	Eppendorf AG, Hamburg, Germany
Thermocycler (Model T3)	Biometra, Goettingen, Germany

Kits/Reagents and Disposables used for RT-PCR.

Table S2

Volume	Reagents
2 µl	RT buffer
2 µl	random primer
0,8 µl	dNTPs
1 µl	RNase Out ™
1 µl	MultiScribe™ Reverse Transcriptase
3,2 µl	DEPC-H_2O

RT-PCR-Master Mix.

Table S3

Temperature	Duration
25 °C	10 min
37°C	120 min
85°C	5 min
4°C	Pause

PCR protocol for RT-PCR. Reaction volume was 20µl per sample (10µl Master Mix and 10µl RNA). cDNA was stored by -20°C.

Table S4

Kits/Reagents and Disposables	Source
PCR System 7500 fast	Applied Biosystems® Life Technologies Darmstadt, Germany
TaqMan® Fast Universal PCR Master Mix	Applied Biosystems® Life Technologies Darmstadt, Germany
TaqMan® Gene Expression Assay	Applied Biosystems® Life Technologies Darmstadt, Germany
HPLC-H_2O	Merck, Darmstadt, Germany

Kits/Reagents and Disposables used for Real Time quantitative PCR.

Table S5

Volume	Reagents
10 µl	TaqMan® Fast Universal PCR Master Mix
1,4 µl	TaqMan® Gene Expression Assay of the examined target gene
7,6 µl	HPLC-H_2O

TaqMan®-PCR reaction solution.

Table S6

Target gene	Synonym	Assay Identification Number
GAPDH	GAPDH	Hs 02758991_g1
HBD1	DEFB 1	Hs 00608345_m1
HBD2	DEFB 4	Hs 00175474_m1
HBD3	DEFB 103	Hs 00218678_m1
HBD4	DEFB 104	Hs 00414476_m1
HD5	DEFA 5	Hs 00360716_m1
HD6	DEFA 6	Hs 00427001_m1
LL-37	CAMP	Hs 00189038_m1

Gene expression assays of the housekeeping gene (GAPDH) and the target genes.

Table S7

Temperature	Duration	Reaction	Cycles
95°C	20 min	First denaturation	
95°C	3 sec	denaturation	45 cycles
60°C	30 sec	annealing/extension	

TaqMan®Real time quantitative PCR protocol. Reaction volume was 20µl per sample (19µl TaqMan®-PCR reaction solution and 1µl cDNA). Negative controls consisted of 19 µl TaqMan®-PCR reaction solution and 1µl HPLC-H_2O.
